# De Novo Transcriptome Assembly and EST-SSR Marker Development and Application in *Chrysosplenium macrophyllum*

**DOI:** 10.3390/genes14020279

**Published:** 2023-01-21

**Authors:** Niyan Xiang, Bojie Lu, Tao Yuan, Tiange Yang, Jiani Guo, Zhihua Wu, Hong Liu, Xing Liu, Rui Qin

**Affiliations:** 1 Laboratory of Extreme Environmental Biological Resources and Adaptive Evolution, Research Center for Ecology, School of Sciences, Tibet University, Lhasa 850000, China; 2 Hubei Provincial Key Laboratory for Protection and Application of Special Plant Germplasm in Wuling Area of China, College of Life Sciences, South-Central Minzu University, Wuhan 430074, China; 3 College of Life Sciences, Zhejiang Normal University, Jinhua 321004, China; 4 State Key Laboratory of Hybrid Rice, Laboratory of Plant Systematics and Evolutionary Biology, College of Life Sciences, Wuhan University, Wuhan 430072, China

**Keywords:** *Chrysosplenium macrophyllum*, transcriptome, EST-SSR, transferability, genetic diversity, population structure

## Abstract

*Chrysosplenium macrophyllum* Oliv., belonging to the family Saxifragaceae, is a traditional and unique Chinese herbal medicine. However, the lack of adequate molecular markers has hampered the progress regarding population genetics and evolution within this species. In this research, we used the DNBSEQ-T7 Sequencer (MGI) sequencing assay to analyze the transcriptome profiles of *C. macrophyllum*. SSR markers were developed on the basis of transcriptomic sequences and further validated on *C. macrophyllum* and other *Chrysosplenium* species. The genetic diversity and structure of the 12 populations were analyzed by using polymorphic expressed sequence tag simple sequence repeat (EST-SSR) markers. A potential pool of 3127 non-redundant EST-SSR markers were identified for *C. macrophyllum* in this study. The developed EST-SSR markers had high amplification rates and cross-species transferability in *Chrysosplenium*. Our results also showed that the natural populations of *C. macrophyllum* had a high level of genetic diversity. Genetic distance, principal component analysis, and popular structure analysis revealed that all 60 samples clustered into two major groups that were consistent with their geographical origins. This study provided a batch of highly polymorphic EST-SSR molecular markers that were developed via transcriptome sequencing. These markers will be of great significance for the study of the genetic diversity and evolutionary history of *C. macrophyllum* and other *Chrysosplenium* species.

## 1. Introduction

*Chrysosplenium* L. is a very small perennial herbaceous genus in the family Saxifragaceae, with tetramerous flowers and petaloid sepals [1]. This genus consists of around 80 species distributed in Asia, Europe, Africa, and America; however, only two species in Chile have been found in the southern hemisphere, and the rest are concentrated in the northern hemisphere [2,3,4,5]. In the northern hemisphere, *Chrysospelnium* species, including ca. 53 species, are mainly distributed in East Asia, with China being one of the diversity centers of this genus, with 39 species, of which 24 are endemic [1,5,6,7]. In accordance with the Flora of China, the literature, and field investigations, *Chrysospelnium macrophyllum* is endemic to China, mainly distributed in 14 Chinese provinces [8,9]. It is a common folk herbal medicine that can treat infantile convulsions, ecthyma, scalds, and lung and ear disorders [10]. Only a few studies have been performed on *C. macrophyllum*, and its chloroplast genomic data have been obtained [11]. Given the lack of rich molecular markers for *C. macrophyllum*, the population structure and genetic diversity of *C. macrophyllum* are still unknown, thus minimizing the exploitation and utilization of this species.

Molecular markers are an extremely popular tool in the analysis of genetic diversity because of their stability, cost-effectiveness, and facile application [12]. The most used molecular markers mainly include restriction fragment length polymorphisms (RFLP), random amplified polymorphic DNA markers (RAPD), amplified fragment length polymorphisms (AFLP), inter simple sequence repeats (ISSR), sequence-related amplified polymorphisms (SRAP), simple sequence repeats (SSR), and single-nucleotide polymorphism (SNP) markers [13,14]. SSRs are the most widely used molecular markers, associated with their codominance, abundance, high polymorphism, good reproducibility, and simple operation [15,16,17]. SSRs can be separated into genomic SSR (gSSR) and expressed sequence tag SSR (EST-SSR) markers, in accordance with their type of sequence source [18]. EST-SSRs have a lower developmental cost than gSSRs and exhibit cross-species transferability and direct correlations with gene functions [18,19]. They have been widely used in plant research, such as studies on *Carex breviculmis* [20], *Pinus koraiensis* [21], *Actinidia eriantha* [22], *Zingiber officinale* [23], *Rosa roxburghii* [24], and *Dendrobium officinale* [25].

Next-generation sequencing technology, especially transcriptome sequencing with Illumina and MGI, is an effective and reliable tool that provides a low-cost means to develop SSR markers [26,27,28,29]. Transcriptome sequencing and *de novo* assembly are essential for studying functional genomics as mining markers, especially markers in non-model organisms that lack sequenced genomes [30,31]. To date, only several nucleotide sequences of *Cymbidium aureobracteatum* have been reported (September 2022), and no *C*. *macrophyllum* ESTs are available in GenBank [32]. In previous studies, only the chloroplast gene *matK* was used to examine the genetic variations of the genus *Chrysosplenium* [33]. However, only a few researchers have investigated *C. macrophyllum*.

In this study, (i) we used the DNBSEQ-T7 Sequencer to obtain the global transcriptome of *C. macrophyllum* and annotated and functionally classified the transcripts. (ii) Then, a number of EST-SSRs were built for *C. macrophyllum* on the basis of these transcripts and we verified their transferability among different *Chrysosplenium* species. (iii) Finally, we evaluated the genetic diversity and structure of 12 populations of *C. macrophyllum*. This study will lay a solid resource foundation for studies on functional genomics, metabolomics, proteomics, and the development and utilization of molecular markers, and also provide important references and new ideas for related studies on the species of *Chrysosplenium*.

## 2. Materials and Methods

### 2.1. Plant Materials, RNA Isolation, and DNA Extraction

The fresh roots, stems, and leaves of *C. macrophyllum* were gathered on 10 August 2021, from Xuanen County, Hubei Province, China, and instantly frozen in liquid nitrogen. Samples were then stored at up to −80 °C until used for RNA isolation. The young leaves of 60 individuals from 12 wild populations of *C. macrophyllum* were collected and placed in sealed bags containing dried silica gel for subsequent DNA isolation. They were collected from seven provinces that included most of the distribution of this species in China (Table 1). The distance between each individual in the population was more than 1 m. Sixteen additional *Chrysosplenium* species were gathered to detected the cross-genome transferability of EST-SSRs (Table 1).

Total RNA was extracted by using the R6827 Plant RNA Kit (Omega Bio-Tek, Inc., Norcross, GA, USA) in accordance with the manufacturer’s instructions. RNA contamination and degradation were supervised with 1% agarose gels. RNA integrity and purity was assayed by using a Qubit 3.0 Fluorometer (Life Technologies, Carlsbad, CA, USA) and NanoDrop One spectrophotometer (NanoDrop Technologies, Wilmington, DE, USA), respectively. Qualified RNA from roots, stems, and leaves of *C. macrophyllum* was mixed in equal amounts for RNA sequencing.

Genomic DNA was extracted by using a modified cetyltrimethylammonium bromide (CTAB) method [34]. DNA integrity and concentration were determined by using 1% agarose gel electrophoresis and NanoPhotometer^®^ NP80 (Implen, München, Germany), respectively. Then, the extracted DNA was diluted with ddH_2_O to the desired working concentration (50 ng/μL) and stored at −20 °C until PCR amplification.

### 2.2. Transcriptome Sequencing and De Novo Assembly

The transcriptome sequencing of *C. macrophyllum* was performed using the DNBSEQ-T7 platform from Wuhan Benagen Technology Co., Ltd. (Wuhan, China). FASTPv0.23.1 [35] was used to remove reads with adaptors, those with more than 5% unknown nucleotides (N), or those with more than 50% low-quality (Q-value 5) bases. Then, the de novo assembly of the high-quality clean reads was conducted by utilizing Trinity v2.8.3 [36] with the parameters of min_contig_length = 500, min_kmer_cov = 3, and min_glue = 15. After assembly, CD-HIT [37] was used for clustering to remove redundant transcripts and unigenes were obtained.

### 2.3. Annotation and Functional Classification

Coding regions within unigenes were detected by using TransDecoder (https://github.com/TransDecoder/TransDecoder/releases, accessed on 10 October 2022), implemented in Trinity software). For the characterization of all the putative functions of the unigenes, the unigenes were compared against public databases, including NCBI nonredundant protein sequences (NR) [38], Kyoto Encyclopedia of Genes and Genomes (KEGG) [39], Gene Ontology (GO) [40], and Clusters of Eukaryotic Orthologous Groups (KOG) (E-value < 1.0 × 10^−5^) [41].

Eggnog-mapper v2 [42] and InterProScan v5.0 (https://github.com/ebi-pf-team/interproscan, accessed on 20 October 2022) were used to obtain GO and KOG annotations. After the prediction of protein sequences, the unigenes were aligned with the NR, Swiss-Prot, and KEGG databases by using Diamond (E-value < 1.0 × 10^−5^) [43].

### 2.4. SSR Identification and Primer Design

The detection and localization of potential SSRs were performed by using the microsatellite tool [44]. The search standards for SSRs were set to the minimum number of 10, 6, 5, 5, 5, and 5 repeat units for mono-, di-, tri-, tetra-, penta-, and hexanucleotide motifs, respectively. Primers for the flanking sequences of the identified microsatellite motifs were designed by using Primer 3 software. The parameters considered for primer designing were as follows: (a) primer length of 18–23 bp with 20 bp as the optimal length; (b) PCR product sizes ranging from 100 bp to 250 bp; (c) GC content ranging from 40% to 60% with the optimum of 50%; (d) annealing temperature between 50 °C and 60 °C with 58 °C as the optimal temperature; and (e) default values for the other parameters.

### 2.5. EST-SSR Validation and Cross-Species Amplification

In total, 58 pairs of primers were randomly chosen and synthesized by Beijing TSINGKE Biological Technology Co., Ltd. (Beijing, China), to develop polymorphic EST-SSR markers. Twelve DNA samples from different populations, including ZJ, BD, HY, NJ, GD, XE, WG, LA, YS, JN, TS, and PA, were used to analyze the primary polymorphisms of the primers. PCR amplification was performed by using BIO-RAD T100 Thermal CyclerTM, and the PCR reaction system was prepared with a 10 μL total reaction volume comprising 5 μL of 2×T5 Super PCR Mix (PAGE) (Beijing TsingKe Biotech Co., Ltd., Beijing, China), 0.4 μL (10 μM) each of the forward and reverse primers, 1 μL of genomic DNA (50 ng/μL), and 3.2 μL of ddH_2_O. The PCR procedure was conducted as follows: an initial denaturation for 2 min at 98 °C; 30 cycles of denaturation at 10 s at 98 °C, annealing at 58 °C for 10 s, and extension at 72 °C for 10 s; and a final extension cycle of 2 min at 72 °C and holding at 4 °C. The amplified PCR products were mixed with 10× loading buffer at the ratio of 1:5 or 1:10 and immediately placed into a mixture of ice water after being denatured at 95 °C for 5 min in a BIO-RAD T100 Thermal CyclerTM. The same denaturation process was performed with PAGE Gel 20 bp ladder marker (Beijing Bio-ulab Biotech Co., Ltd., Beijing, China) as the molecular size standard. Then, the mixture of PCR products and 10× loading buffer was subjected to 6% denatured polyacrylamide gel electrophoresis at 90 W for 1–1.5 h and visualized by using silver nitrate staining.

After the screening of polymorphic primers, 39 pairs of primers with the expected band sizes were selected for cross-species amplification validation on other *Chrysosplenium* species. The PCR reaction system and conditions were the same as above. After PCR amplification was completed, gel electrophoresis was performed utilizing 3% agarose. Moreover, 50 bp DNA Ladder was used as a marker to determine the size of PCR products. Agarose gel photographs were taken using an automated gel imaging system. Then, 10 pairs of polymorphic primers were further selected for the analysis of genetic diversity in 60 individuals from 12 *C. macrophyllum* populations. The PCR amplification conditions and genotyping methods were the same as those above. The PCR bands of gel images observed under a light lamp were marked as present (1) or absent (0).

### 2.6. SSR Data Analysis

GENODIVE version 3.06 [45], which can handle genetic data from polyploids or mixed-ploidy datasets, was used to calculate the following population genetic parameters: the number of alleles (*Na*), effective number of alleles (*Ne*), observed (*Ho*) and expected (*He*) heterozygosity, and inbreeding coefficient (*Fis*). The *Ho* and *He*, polymorphic information content (PIC), and Shannon diversity index (*I*) of each population and locus were estimated by using POLYGENE v1.2 [46]. Differentiation between *C. macrophyllum* populations was assessed on the basis of G_ST_. Analysis of molecular variance (AMOVA) was performed by using POLYGENE v1.2 to obtain the genetic variation among populations.

A neighbor-joining tree based on *D*_A_ genetic distance was established for *C. macrophyllum* individuals by using POPTREE v.2 software [47]. Principal coordinate analysis (PCoA) was performed with Cavalli–Sforza’s chord distances, which have been shown to be the least biased distance measure in the absence of dosage information [48]. STRUCTURE version 2.3.4 [49] was used to infer the population structure using an admixture model with correlated allele frequencies. The potential number of genetic clusters (K) ranged from 1 to 10, and 10 independent replicates were run for each K value with a 100,000 burn-in period and 1,000,000 Markov chain Monte Carlo iterations. The online program STRUCTURE HARVESTER [50] was used to infer the optimal K in accordance with the method of Evanno et al. [51]. The program CLUMPP version 1.1.2 [52] was applied to estimate the averaged admixture coefficients for each K value. The clustering results were visualized by using Distruct version 1.1 [53].

## 3. Results

### 3.1. De Novo Assembly of the Transcriptome

**Figure 1:**
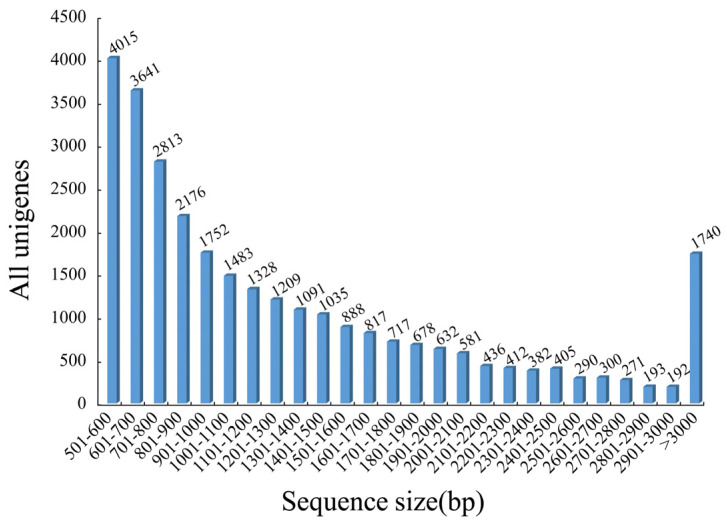
Distribution of unigene lengths of *C. macrophyllum*.

After adapter removal and low-quality sequence filtering, 40,507,062 high-quality clean reads were obtained. The Q30 base percentage reached 93.00%, and the GC content was 42.00%. Then, 63,961 assembled transcripts with the mean length of 1551.85 bp, GC content of 40.21%, and N50 length of 1901 bp were generated by using Trinity v2.8.3. Subsequently, the longest copy of assembled transcripts isomer was extracted. After redundancy removal, the longest remaining transcripts were regarded as unigenes. Finally, a total of 29,477 unigenes with the mean length of 1341.32 bp, the maximum length of 23,968 bp, and N50 of 1646 bp (Table 2) were obtained. A total of 14,397 unigenes (48.84%) had lengths less than 1000 bp; 9878 unigenes (33.51%) had lengths between 1001 and 2000 bp; and 5202 unigenes (17.65%) had lengths > 2000 bp (Figure 1).

### 3.2. Gene Annotation Based on Different Databases

A total of 15,647 protein-coding unigenes were predicted by using TransDecoder and submitted to the NR, KOG, Swiss-Prot, KEGG, and GO databases for functional annotation. As shown in Table 3, 11,115 unigenes were successfully annotated, including 10,946 (37.13%) in NR, 6670 (22.63%) in KOG, 8422 (28.57%) in Swiss-Prot, 2021 (6.85%) in KEGG, and 7836 (26.58%) in GO.

**Figure 2:**
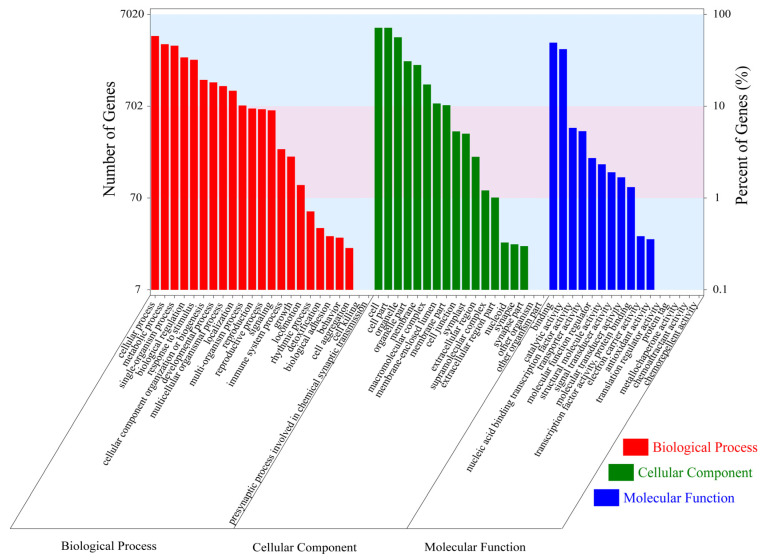
GO classification of *C. macrophyllum*.

On the basis of functional annotation, the unigenes were divided into three main GO categories (biological process, molecular function, and cellular component) and 57 subcategories (Figure 2). In the biological process category, “cellular process” was the largest subgroup, followed by “metabolic process”, “single-organism process”, and “biological regulation”. Among the 18 different cellular component categories for *C. macrophyllum* unigenes, the categories “cell” and “cell part” were the most abundant. The molecular function category contained 16 GO terms, among which “binding”, “catalytic activity”, and “nucleic acid binding transcription factor activity” were highly represented.

**Figure 3:**
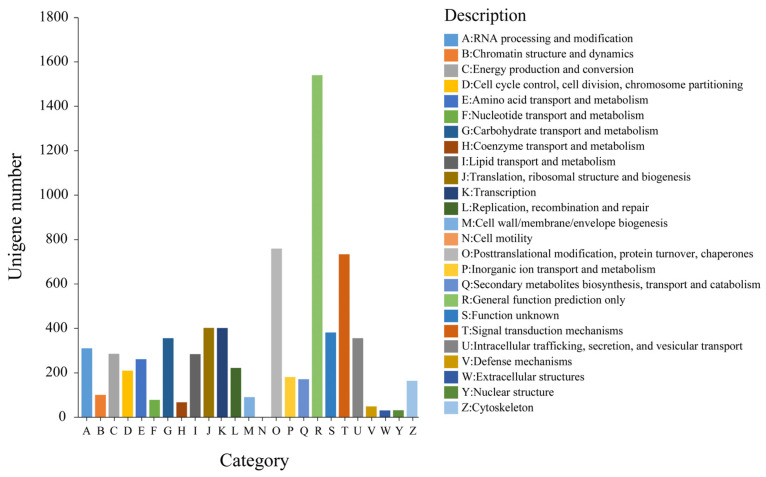
KOG classification of *C. macrophyllum*.

The unigenes were annotated and functionally classified into 25 KOG categories, and a large number of the unigenes were assigned to more than one category (Figure 3). Among these categories, “general function prediction only” (1541, 23.10%) was the most dominant. “Post-translational modification, protein turnover, chaperones” (759, 11.38%) constituted the second-largest cluster, which was followed by “signal transduction mechanisms” (734, 11.00%). However, only two unigenes were annotated to “cell motility” (Figure 3).

**Figure 4:**
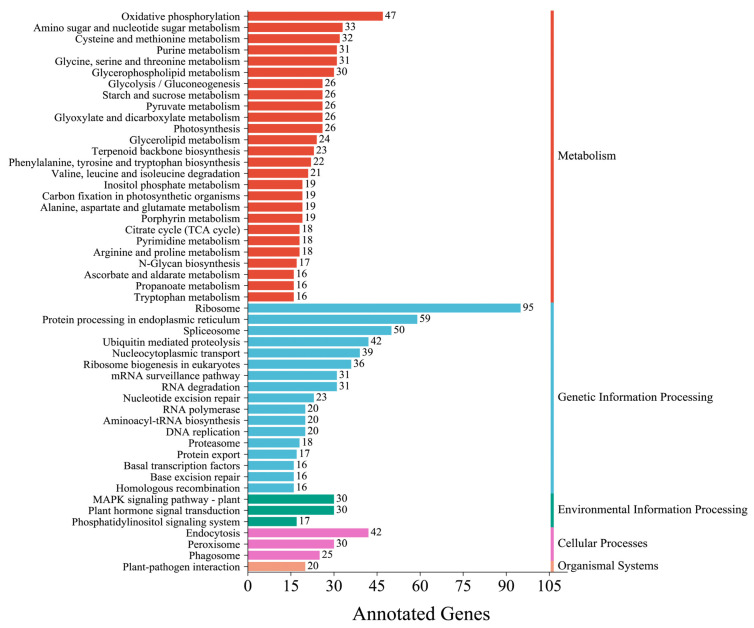
KEGG classification of *C. macrophyllum*.

A total of 2020 unigenes were found in the KEGG database and assigned to 127 KEGG functional pathways belonging to five large groups (“metabolism”, “genetic information processing”, “environmental information processing”, “cellular processes”, and “organismal systems”). “Ribosome” (95), “protein processing in endoplasmic reticulum” (59), and “spliceosome” (50) were the main pathways among the top 50 pathways (Figure 4). In addition, 23 unigenes were found in the “terpenoid backbone biosynthesis” pathway.

**Figure 5:**
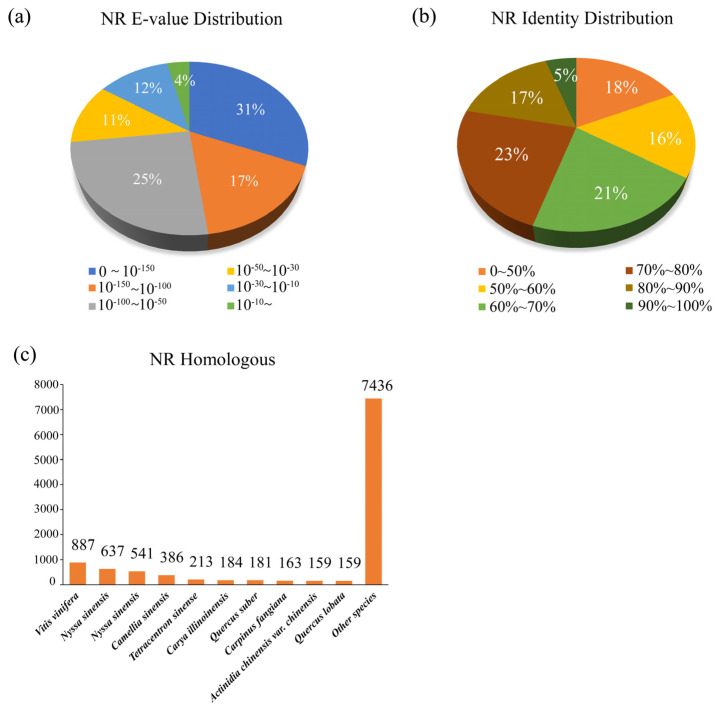
Homology searches of *C. macrophyllum* unigene and characteristics of non-redundant protein databases (Nr). (**a**) The E-value distribution of unigene BLASTx hits for every assembly. (**b**) BLASTx hit profiles for every assembly of unigenes. (**c**) Distribution of accessions hit by BLASTx at the top of each assembly of unigenes.

The E-value distribution revealed that 31.26% of the unigenes yielded significant hits in the NCBI NR nucleotide database (Figure 5a), and approximately 21.65% of these unigenes exhibited greater than 80% identity (Figure 5b). The sequence alignment results of the NR protein revealed that 887 unigenes could be aligned with *Vitis vinifera*, 637 unigenes could be aligned with *Nyssa sinensis*, and 541 could be aligned with *Vitis riparia* (Figure 5c).

### 3.3. Frequency and Distribution of SSRs

A total of 5573 unigenes containing 6985 SSRs were identified among 29,477 unigenes by using MISA software. Among these unigenes, 1091 contained more than one SSR, and 500 SSRs presented a compound formation. In *C. macrophyllum*, the SSR motif were found to be distributed every 5.67 kb on average (Table 4). In the identified SSRs, the mono-nucleotide motifs were the most enriched, with a proportion of 46.09%, followed by di- (33.34%), tri- (19.18%), tetra- (0.82%), penta- (0.17%), and hexa- (0.40%) nucleotide motifs (Table 4). A total of 67 different repeat motifs were found in all SSR loci. A/T (3170) was the dominant motif in mononucleotide repeats, accounting for 45.38% of all motifs. The next most dominant motif was C/G (49), which accounted for 0.70% of all motifs. Among the dinucleotide repeats, the most frequent motifs were AG/CT (1433, 20.52%), followed by AT/AT (788, 11.28%), AC/GT (106, 1.52%), and CG/CG (2, 0.03%). Ten different trinucleotide repeat motifs were identified, among which ATC/ATG, AAG/CTT, AAC/GTT, and AGG/CTT accounted for 4.31%, 4.27%, 2.25%, and 2.22%, respectively, on the basis of frequency. The frequencies of the other six motifs were less than 2%. The frequencies of tetranucleotide repeats were 0.82%, and the total frequencies of pentanucleotide and hexanucleotide repeats were 0.57% (Table 5).

### 3.4. Development and Transferability of EST-SSR Markers

A total of 3127 pairs of primers were successfully designed on the basis of the 6985 SSRs. Of these, 58 pairs, mainly comprising dinucleotide and trinucleotide repeat units, were selected for amplification and polymorphism assessment. The results showed that 39 (67.24%) primers generated the expected size bands, including six pairs of monomorphic primers and 33 pairs of polymorphic primers. Finally, 10 highly polymorphic primers were selected to analyze the genetic diversity of 60 *C. macrophyllum* samples from 12 populations.

Whether the primer pairs designed from the EST sequences of *C. macrophyllum* could also effectively amplify the same SSR motifs in 16 *Chrysosplenium* species was verified. Of the 39 EST-SST primers with the expected band size, only three (7.69%) successfully amplified SSR motifs in all *Chrysosplenium* species, whereas 33 resulted in amplification in some but not all species, and three failed to result in amplification in all 16 additional species (Table S1). The top three species with the highest success rates in cross-amplification trials were *C. hydrocotylifolium* (79.49%), *C. lanuginosum* (64.10%), and *C. nudicaule* (61.54%).

### 3.5. Genetic Diversity and Structure

By using the set of 10 SSRs, 94 alleles were detected across the 60 *C. macrophyllum* samples for an average of 9.4 alleles per locus. The minimum number of alleles detected at each locus was five (CsSSR30) and the maximum number was 15 (CsSSR5). The PIC values ranged from 0.565 (CsSSR44) to 0.855 (CsSSR5), with the average of 0.678 (Table S2). The values of genetic diversity at the population level are shown in Table 6. *Ne* ranged from 1.899 in YS to 3.513 in BD, averaging 2.699 alleles per population. *Ho* as estimated by GenoDive ranged from 0.480 (LA) to 0.717 (JN), whereas the *Ho* estimated by using Polygene ranged from 0.265 (LA) to 0.710 (JN). *He* calculated by GenoDive and Polygene ranged from 0.459 (YS) to 0.393 (BD and TS) and 0.392 (LA) to 0.636 (BD), respectively. The observed gene heterozygosity was lower than the expected gene heterozygosity. The *Fis* of BD, NJ, LA, and TS was greater than 0, whereas that of the other populations was less than 0. The overall G_ST_ among all populations was 0.218. Pairwise comparisons of genetic differentiation between populations indicated that G_ST_ ranged from 0.043 (between populations ZJ and HY) to 0.249 (between populations PA and HY) (Table S3). AMOVA revealed that the genetic variation within populations (65.22%) was higher than that among populations (34.78%) of *C. macrophyllum*, suggesting a high level of differentiation (Table S4).

**Figure 6:**
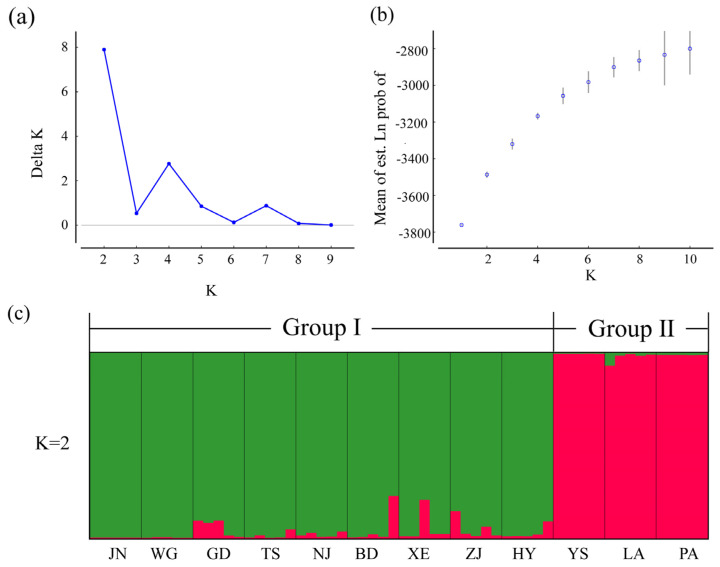
Structure analysis of 60 *C. macrophyllum* from 12 populations based on 10 EST-SSRs. (**a**) Distribution of ΔK in STRUCTURE analysis. (**b**) The likelihood L(K) values presented for K = 1–10. (**c**) Histogram of the STRUCTURE analysis for the model with K = 2 (showing the highest ΔK). Different colors represent genetic stock.

**Figure 7:**
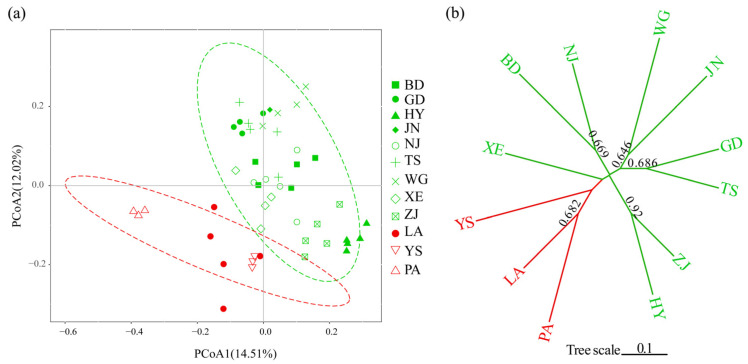
Graphical representation of differentiation between populations. (**a**) Principal coordinate analysis (PCoA). (**b**) Neighbor-joining phylogenetic tree of 60 *C. macrophyllum* individuals.

The population structure of *C. macrophyllum* was analyzed by using STRUCTURE 2.3.4, and the optimal K value was observed at K = 2, with the maximum ΔK value (Figure 6a,b). All collected individuals were divided into two genetic groups (Figure 4c). Group I contained eight populations (JN, WG, GD, TS, NJ, BD, XE, ZJ, and HY), whereas Group II included three populations (YS, LA, and PA) (Figure 6c). PCoA based on the 10 EST-SSR markers was used to evaluate the population genetic structure. Consistent with the results of structure analysis, the PCoA results also revealed two groups based on genetic distance (Figure 7a). The first and second axes explained 14.51% and 12.02% of the total variation, respectively. In addition, a neighbor-joining tree was constructed by using *D*_A_ distances. In the tree, individuals were divided into two groups, in agreement with the two genetic groups identified by PCoA and STRUCTURE (Figure 7b).

## 4. Discussion

Progress in studies on *C. macrophyllum* has been very slow compared with that in studies on other model plants with a reference genome. Access to genomic data is crucial for comprehending and expanding the study of a species. Transcriptome sequencing is more affordable and suitable for studying the genomes of non-model plant species than whole-genome sequencing [54]. In this study, the transcriptome sequencing of *C. macrophyllum* generated 40,507,062 high-quality clean reads (93.00% Q30), which were assembled into 29,477 non-redundant unigenes with an N50 of 1646 bp and an average length of 1341.32 bp. The current results were comparatively better than those previously reported for *Actinidia eriantha* (average length = 594 bp, N50 = 973 bp) [22] and *Panax vietnamensis* (average length = 598.32 bp, N50 = 942 bp) [55] and similar to those reported for *Pistacia chinensis* (average length = 1325 bp, N50 = 2027 bp) [56] and *P. vietnamensis* var. *fuscidicus* (average length = 1304 bp, N50 = 2108 bp) [57]. Compared with *C. aureobracteatum* (70,753,963 bp total assembled bases), we obtained more assembled bases in *C. macrophyllum* (99,257,989 bp total assembled bases) [32]. These findings indicated that the quality of sequencing and assembly was high and can meet the requirements of subsequent transcriptomic data analysis.

Among the 29,477 unigenes, 11,478 (38.94%) were successfully annotated in the public protein databases of NR, KOG, Swiss-Prot, KEGG, and GO. The annotated unigenes could provide valuable information for future studies on *C. macrophyllum*. The remaining unmatched unigenes in the protein databases may be incomplete sequences lacking key information for annotation and/or the genes specific to *C. macrophyllum* without previous characterization. The BLASTX search against the NR database revealed that although only 7.83% of the identified unigenes of *C. macrophyllum* were similar to those of *V. vinifera*, it was the species with the largest number of hits for *C. macrophyllum* unigenes. In fact, *C. macrophyllum* and *V. vinifera* are members of Saxifragaceae and Vitaceae, respectively, and are therefore genetically and evolutionarily distant from each other. This result may be attributed to the lack of whole-genome sequences for any species of Saxifragaceae in public databases. The division of the identified unigenes into 25 subterms and 57 subcategories in the GO and KOG databases suggested that the annotated unigenes have a wide range of important functions in *C. macrophyllum*. A total of 2020 unigenes were mapped to 127 biological pathways, among which the metabolism category was the largest, followed by the genetic information processing category. These data revealed the active metabolic processes and the synthesis of various metabolites. In *C. nudicaule*, *C. carnosum*, and other *Chrysosplenium* species, flavonoids and triterpenoids are the main active components; these components help in resistance against biological and environmental stresses, such as cold, drought, and pests [10,58,59]. In this study, we recorded the unigenes for the terpenoid backbone biosynthesis pathway.

In this study, 5573 unigene genes contained 6985 SSR loci with the distribution frequency and density of 23.46% and 5.67 kb, respectively. The rate of distribution frequency found in this work was higher than that reported for *Epimedium sagittatum* (3.67%) [60] and *Phyllostachys violascens* (13.83%) [17] but lower than that reported for *Phoebe bournei* (55.57%) [61]. The abundance and distribution of SSRs are influenced by numerous factors, including species differences, SSR search criteria, dataset size, SSR development tools, and sequence redundancy [56,62,63]. The SSR types in the transcriptome of *C. macrophyllum* were relatively abundant, ranging from mononucleotide repeats to hexanucleotide repeats. Consistent with the EST-SSR distribution reported in *C. aureobracteatum* [32], the dinucleotide (33.34%) and trinucleotide (19.18%) repeats became dominant when mononucleotides were excluded. Of the mononucleotide motifs, A/T (45.38%) motifs were far more abundant than the G/C (0.70%) motif, as in most plants [64]. Among dinucleotide repeats, AG/CT (13.97%) was the most abundant; this result was identical to previous findings on monocots and eudicots [65,66]. AT/TA (6.09%) and AC/GT (2.21%) were the next most abundant motifs. In *C. macrophyllum*, the most predominant trinucleotide repeat motif was ATC/ATG (4.31%), followed by AAG/CTT (4.27%). In contrast to those in *C. macrophyllum*, the most frequent trinucleotide repeat motifs were AGG/CCT in *Z. officinale* [23], AAG/CTT in *E. sagittatum* [60], and CCG/GGC in *Elymus sibiricus* [67]. Previous studies on other species indicated that the trinucleotide motif AAG/CTT is a major motif and that CCG/CGG is a rare motif in dicotyledonous plants, but is a common motif in monocots [68]. In this study, the trinucleotide CCG/CGG motif (0.30%) was the least abundant trinucleotide repeat, likely due to the high GC content and consequent codon usage bias in monocots [69,70].

We successfully designed 3127 (44.77%) primer pairs out of 8658 EST-SSR candidate loci. The failure of primer design for the remaining SSR loci may be due to the short flanking sequences of the SSR loci or the inappropriate motif of the required SSR markers. Among the 58 primer pairs selected, 39 (67.24%) resulted in successful amplification in *C. macrophyllum*, among which 33 (56.90%) were polymorphic. The rate of polymorphism in this species was lower than in *Vigna mungo* (58.2%; n = 18) [71] but higher than in *R. roxburghii* (29.4%; n = 16) [24]. Therefore, in this study, the rate of EST-SSR polymorphisms was relatively high. The transferability of markers corresponds to the similarity of genomes, which can reflect the genomic relationships and even the evolutionary relationships between species [72]. In general, close genetic relationships among different species are expected with the high transferability of EST-SSR markers. In this study, the transferability of the 39 EST-SSRs from *C. macrophyllum* to *C. hydrocotylifolium* was the highest, suggesting that *C. macrophyllum* had a closer relationship with *C. hydrocotylifolium* than with other *Chrysosplenium* species. This result was consistent with the close phylogenetic relationship between the two species [5]. Significantly, only 3 (7.69%) out of 39 EST-SSR markers failed to amplify successfully in all 16 *Chrysosplenium* species. The high transferability of the markers indicated that the flanking sequences of EST-SSRs were highly conserved among related species. These results suggest that the markers developed in our study may provide a powerful molecular tool for the evolutionary adaptation and phylogenetic analyses of *C. macrophyllum* and other species of *Chrysosplenium*.

In this study, the samples were subdivided into two main groups on the basis of STRUCTURE analysis, and the phylogenetic analysis of the NJ tree and PCoA analysis supported the two genetic clusters. The species from the YS, LA, and PA populations were allocated into one cluster, and geographically originated from the Ta-pieh Mountains, Tianmu Mountains, and Dapan Mountains, respectively. The classification of species from the same area into one group is correlated with the geographical distribution and environmental conditions. Geographic isolation may have contributed to the genetic differences. In addition, the population structure, NJ tree, and PCoA based on the genotypic data clearly showed obvious genetic differentiation among *C. macrophyllum* species. The set of EST-SSRs obtained in this work would facilitate the diversity analysis of *C. macrophyllum*.

## 5. Conclusions

The de novo transcriptome sequencing of *C. macrophyllum* was performed by using the DNBSEQ-T7 sequencing platform. We obtained a large number of ESTs and identified 6985 EST-SSRs. Our results provided a potential pool of 3127 non-redundant EST-SSR markers for *C. macrophyllum*. The developed EST-SSR markers had high amplification rates and cross-genome transferability of various *Chrysosplenium* species. Furthermore, 10 EST-SSR markers were used to analyze the genetic diversity of 60 *C. macrophyllum* individuals. Our results showed that the populations of *C. macrophyllum* had a high level of genetic diversity. Cluster analysis demonstrated that all 60 individuals clustered into two groups, mainly in accordance with their origins. These transcriptome data will provide genetic resources for the functional study of *C. macrophyllum*. The numerous EST-SSR markers developed in this study represent a valuable tool for the genetic diversity and evolutionary analyses of *C. macrophyllum* and other *Chrysosplenium* species.

## Figures and Tables

**Table 1:** Characteristics of analyzed *Chrysosplenium* species in this study.

Species	Location	Latitude (N)/Longitude (E)	Elevation (m)	Sample Size	Voucher
*C. macrophyllum*	Zhijin, Guizhou	N: 26°39′03″/E: 105°34′29″	1950	5	–
	Badong, Hubei	N: 31°15′30″/E: 110°23′01″	1425	5	HSN6460
	Hongya, Sichuan	N: 29°30′26″/E: 103°15′24″	1770	5	–
	Nanjiang, Sichuan	N: 32°41′18″/E: 106°47′47″	1440	5	–
	Guidong, Hunan	N: 25°59′38″/E: 113°43′19″	1220	5	–
	Xuanen, Hubei	N: 30°01′35″/E: 109°43′13″	1164	5	HSN5500
	Wugang, Hunan	N: 26°38′58″/E: 110°36′46″	1030	5	–
	Linan, Zhejiang	N: 30°20′14″/E: 119°26′03″	770	5	–
	Yinshan, Hubei	N: 30°58′05″/E: 116°01′37″	740	5	–
	Jianning, Fujian	N: 26°47′04″/E: 116°56′04″	690	5	–
	Tongshan, Hubei	N: 29°21′53″/E: 114°34′06″	590	5	HSN13118
	Panan, Zhejiang	N: 28°57′43″/E: 120°33′42″	510	5	–
*Chrysosplenium ramosum*	Fusong, Jilin	N: 42°10′27″/E: 127°30′30″	400	1	SJH2017052107372
*Chrysosplenium serreanum*	Fusong, Jilin	N: 42°10′32″/E: 127°29′03″	412	1	SJH2017052107371
*Chrysosplenium japonicum*	Hangzhou, Zhejiang	N: 30°15′02″/E: 120°6′59″	19	1	HSN7909
*Chrysosplenium griffithii* var. *intermedium*	Kangding, Sichuan	N: 30°06′30″/E: 101°48′06″	3640	1	HSN09825
*Chrysosplenium glossophyllum*	Doujiangyan, Sichuan	N: 30°55′49″/E: 103°28′54″	1049	1	QCS2017102608035
*Chrysosplenium alternifolium*	Fusong, Jilin	N: 42°10′27″/E: 127°30′30″	400	1	SJH2017052107369
*Chrysosplenium microspermum*	Nanchuan, Chongqing	N: 29°01′02″/E: 107°11′32″	1987	1	–
*Chrysosplenium giraldianum*	PingWu, Sichuan	N: 32°53′19″/E: 104°09′50″	2430	1	JZ2018042507981
*Chrysosplenium qinlingense*	PingWu, Sichuan	N: 32°53′19″/E: 104°09′50″	2430	1	HSN7980
*Chrysosplenium lectus-cochleae*	Fusong, Jilin	N: 42°10′27″/E: 127°30′30″	400	1	HSN7379
*Chrysosplenium axillare*	Tianzhu, Gansu	N: 37°03′38″/E: 102°46′06″	3275	1	–
*Chrysosplenium forrestii*	Gongshan, Yunnan	N: 28°04′25″/E: 98°45′09″	3900	1	HSN7797
*Chrysosplenium lanuginosum*	Badong, Hubei	N: 31°21′49″/E: 110°23′17″	1777	1	BD2017030507343
*Chrysosplenium delavayi*	Quanzhou, Guangxi	N: 25°40′12″/E: 111°3′16″	250	1	–
*Chrysosplenium hydrocotylifolium*	Quanzhou, Guangxi	N: 25°40′10″/E: 111°3′16″	280	1	–
*Chrysosplenium nudicaule*	Chayu, Xizang	N: 28°36′57″/E: 98°03′37″	4426	1	–

**Table 2:** Summary of the de novo assembly of *C. macrophyllum*.

Category	Items	Number
Raw Reads	Total raw reads	40,782,638
Clean Reads	Total clean reads	40,507,062
	Total clean nucleotides (nt)	6,052,073,283
	Q30 (%)	93.00%
	N (%)	0%
	GC (%)	42.00%
Trancripts	Total trinity transcripts	63,961
	Total trinity genes	29,508
	GC (%)	40.21
	N50 (bp)	1901
	Maximum length (bp)	23,968
	Mean length (bp)	1551.85
	Total assembled bases	99,257,989
Unigenes	Total unigenes	29,477
	GC (%)	40.06
	N50 (bp)	1646
	Maximum length (bp)	23,968
	Mean length (bp)	1341.32
	Total assembled bases	39,538,014

**Table 3:** Functional annotation of *C. macrophyllum* in different databases.

Category	Number	Percentage (%)
Nr annotation	10,946	37.13
KOG annotation	6670	22.63
Swiss-prot annotation	8422	28.57
KEGG annotation	2020	6.85
GO annotation	7836	26.58
All annotated unigenes	11,115	37.71

**Table 4:** Sequence searching for the SSR markers of *C. macrophyllum*.

Searching Item	Number
Total number of identified SSRs	6985
Number of SSR-containing sequences	5573
Number of sequences containing more than 1 SSR	1091
Number of SSRs present in compound formation	500
Frequency of SSR	1/5.67 kb
Mononucleotide	3219
Dinucleotide	2329
Trinucleotide	1340
Tetranucleotide	57
Pentanucleotide	12
Hexanucleotide	28

**Table 5:** Frequencies of different repeat motifs in SSRs of *C. macrophyllum*.

Repeats	5	6	7	8	9	10	11	12	13	14	15	16+	Total	Percentage (%)
A/T						1780	499	248	132	92	72	347	3170	45.38
C/G						3	7	3	8	5	2	21	49	0.70
AC/GT		64	27	8	3	1	1	1				1	106	1.52
AG/CT		788	338	148	64	35	22	11	3	8	4	12	1433	20.52
AT/AT		261	166	107	82	49	44	79					788	11.28
CG/CG		2											2	0.03
AAC/GTT	107	31	9	6	1		1		1			1	157	2.25
AAG/CTT	195	67	17	10		1		3		2		3	298	4.27
AAT/ATT	76	22	14	4		2	1	1			1	2	123	1.76
ACC/GGT	114	22	14	5									155	2.22
ACG/CGT	30	1											31	0.44
ACT/AGT	41	11	8	2	1	1							64	0.92
AGC/CTG	84	30	4	3				1		1			123	1.76
AGG/CCT	46	13	4	3		1							67	0.96
ATC/ATG	209	50	20	18	1	1	1		1				301	4.31
CCG/CGG	17	4											21	0.30
AAAC/GTTT	1			1	1	1							4	0.06
AAAG/CTTT	1												1	0.01
AAAT/ATTT	18	1											19	0.27
AACC/GGTT												1	1	0.01
AACG/CGTT	1			1									2	0.03
AAGG/CCTT	1												1	0.01
AATC/ATTG	4	1											5	0.07
AATG/ATTC		1	1										2	0.03
AATT/AATT	2												2	0.03
ACAT/ATGT	4												4	0.06
ACCC/GGGT	2												2	0.03
ACCT/AGGT	3												3	0.04
ACTC/AGTG		2											2	0.03
AGAT/ATCT	2	1		1									4	0.06
AGCT/AGCT	1												1	0.01
AGGG/CCCT	1												1	0.01
ATCG/ATCG	3												3	0.04
Other	11	12	1	6	2	0	2	0	0	1	1	4	40	0.57
Total	974	1384	623	323	155	1875	578	347	145	109	80	392	6985	100

**Table 6:** Genetic diversity within *C. macrophyllum* populations at 10 SSR markers.

Population	GenoDive					Polygene			
	*Na*	*Ne*	*Ho*	*He*	*Fis*	*Ho*	*He*	PIC	*I*
ZJ	4.200	3.034	0.716	0.675	−0.060	0.613	0.591	0.526	1.098
BD	5.400	3.513	0.655	0.693	0.055	0.648	0.636	0.600	1.314
WG	3.800	3.003	0.650	0.631	−0.029	0.644	0.591	0.536	1.089
HY	3.300	2.544	0.640	0.620	−0.032	0.592	0.551	0.483	0.954
NJ	4.200	3.042	0.567	0.659	0.141	0.558	0.590	0.547	1.138
GD	3.900	2.852	0.695	0.677	−0.026	0.684	0.624	0.554	1.113
JN	2.600	2.600	0.717	0.628	−0.141	0.710	0.583	0.484	0.913
PA	2.000	2.000	0.508	0.482	−0.056	0.500	0.425	0.333	0.624
LA	2.200	1.909	0.480	0.530	0.094	0.265	0.392	0.321	0.605
YS	2.000	1.899	0.513	0.459	−0.118	0.435	0.408	0.322	0.599
TS	3.800	2.853	0.688	0.693	0.006	0.636	0.626	0.560	1.117
XE	3.900	3.141	0.691	0.682	−0.013	0.662	0.624	0.575	1.157

## Data Availability

Transcriptome data created from this research submitted to NCBI under the SRA ID (SRR23148709), BioProject ID (PRJNA925660) and BioSample ID (SAMN32803129).

## Supplementary Materials

The following supporting information can be downloaded at: https://www.mdpi.com/article/10.3390/genes14020279/s1, Table S1: Cross-species amplification of the 39 microsatellite loci in *Chrysosplenium*; Table S2: Characteristics of polymorphic SSR loci tested in 60 individuals of *C. macrophyllum*; Table S3: Nei’s genetic distance among 12 populations of *C. macrophyllum*; Table S4: Analysis of molecular variance for *C. macrophyllum* populations.
